# Epidemiology and Risks Survey of *Onchocerca volvulus* Infection in Igbo-Eze North Local Government Area, Enugu State, Nigeria

**DOI:** 10.3390/tropicalmed10100285

**Published:** 2025-10-06

**Authors:** Ifeoma Esther Aniaku, Grace Chinenye Onyishi, Chigozie Godwin Nwosu, Godwin Ikechukwu Ngwu, Chioma Janefrances Okeke, Uche Boniface Oraneli, Chidiebere Agha Otuu, Nicholas Arome Akobe, Augustine Uchechukwu Nnama, Kyrian Ikenna Onah

**Affiliations:** 1 Parasitology and Public Health Unit, Department of Zoology and Environmental Biology, University of Nigeria, Nsukka 410105, Enugu State, Nigeria; aniakuesther@gmail.com (I.E.A.); grace.onyishi@unn.edu.ng (G.C.O.); godwin.ngwu@unn.edu.ng (G.I.N.); oranelibonifaceuche@gmail.com (U.B.O.); akobenicholas@gmail.com (N.A.A.); uchechukwu.nnama.pg02374@unn.edu.ng (A.U.N.); kyrian.onah.pg90901@unn.edu.ng (K.I.O.); 2 Kemak Foundation, Abakaliki 480261, Ebonyi State, Nigeria; 3 Department of Zoology, Nnamdi Azikiwe University, Awka 420007, Anambra State, Nigeria; chioma450@gmail.com; 4 Parasitology, Public Health and Epidemiology Unit, Department of Animal and Environmental Biology, Federal University Oye-Ekiti, Oye 370112, Ekiti State, Nigeria; chidiebere.otuu@fuoye.edu.ng

**Keywords:** onchocerciasis, prevalence, risk factors, Igbo-Eze North, Enugu State

## Abstract

**Background:** An epidemiological survey of *Onchocerca volvulus* infection and onchocerciasis in Igbo-Eze North Local Government Area, Enugu State, Nigeria, was undertaken to assess its distribution and risks among individuals in the area. **Methods:** A total of 201 residents who have lived in the study area for at least one year were recruited. At recruitment, they were examined using a rapid assessment method. Their demographic information and risk factors were obtained using a structured questionnaire. **Results:** The overall prevalence for onchocerciasis was 3.5% (7/201). The prevalence of onchocerciasis was observed to be non-significantly (*p* = 0.689) different among the studied communities. The sex and age-related prevalence differences were non-significant (*p* > 0.05); however, onchocerciasis was more prevalent among males and those between 30 and 39 years of age. The prevalence of onchocerciasis was higher (*p* = 0.001) among farmers/fishermen. The significant risk associated with onchocerciasis is the proximity of the water body to houses (*p* = 0.034). **Conclusions:** The onchocerciasis prevalence was low and not dependent on sex or age but rather occupation. Risks to onchocerciasis are environmental and occupational, with chances of scaled up prevalence and burden overtime if unchecked. There is a need for awareness campaigning to enable proper education of the people about onchocerciasis in the area and neighboring communities.

## 1. Background

The arthropod vector *Simulium* transmits *Onchocerca volvulus* [1]. The filarial nematode *Onchocerca volvulus* is transmitted from person to person by the repeated bites of infected black flies. Nematode pathogenesis is associated with host inflammation invoked by living or dead parasites [1]. According to Ubachukwu [2], onchocerciasis presents a horrible picture to behold, with a poor prognosis, as well as inflicts tremendous economic, psychological and socioeconomic damage on individuals and entire communities in areas where it is endemic. The most serious lesions are those of the eye, which may lead to partial or total loss of vision. People with onchocercal skin disease are stigmatized in their communities; this condition limits the range of social involvement and can affect the sexual life of affected individuals. The work of Okoye et al. [3] on the assessment of onchocerciasis prevalence in Etteh, Igbo-Eze North LGA, Enugu State, after a decade of mass mectizan chemotherapeutic intervention revealed that despite the long annual period of free distribution of mectizan in the area, onchocerciasis prevalence was still high. Also, Ekpo et al. [4] reported high nodule and microfilariae prevalence in studied villages in Enugu State, which ranged from 42 to 66.7% and 32 to 51.1%, respectively. Hence, the need for continuous surveillance becomes paramount. Our study investigated *Onchocerca volvulus* infection and onchocerciasis and as well as its risks in Igbo-Eze North Local Government Area of Enugu State, South-Eastern Nigeria.

## 2. Methods

### 2.1. Study Area

**Figure 1:**
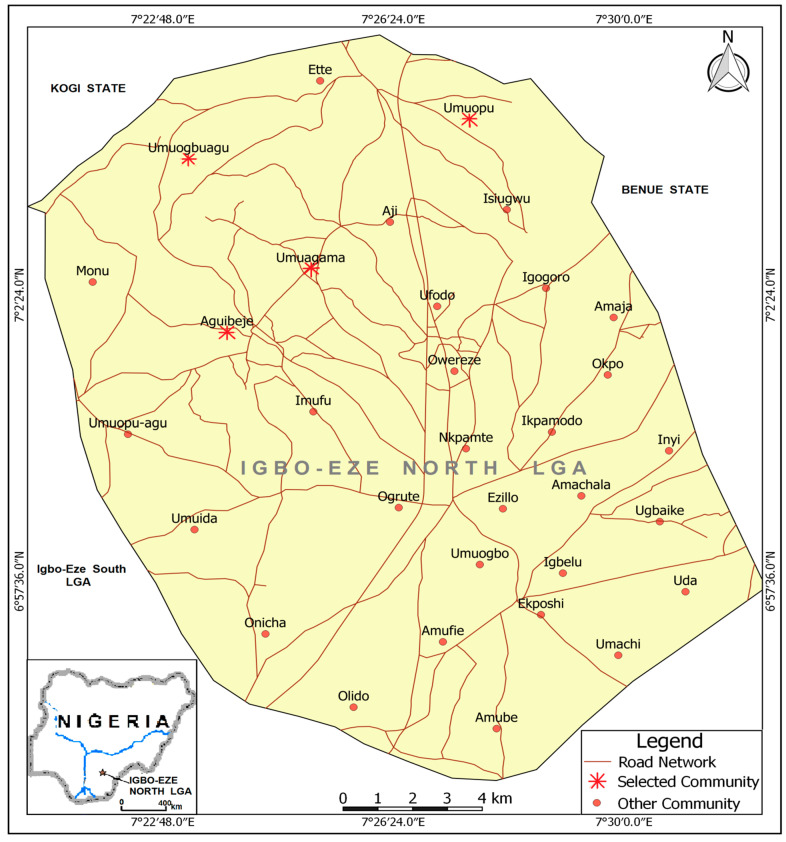
Map of Igbo-Eze North Local Government Area showing the selected communities. **Source**: GIS Unit, Department of Geography, University of Nigeria Nsukka.

The study was carried out in Igbo-Eze North Local Government Area located in the northern part of Enugu State, Nigeria. Igbo-Eze North Local Government Area (Figure 1) is made up of two clans, namely, Enugu Ezike and Ette. Ette is a far smaller town, while Enugu Ezike accounts for over 90% of the population of Igbo-Eze North Local Government Area. Ogrute in Enugu Ezike, a semi-urban area, is the administrative headquarters. The Local Government has an area of 293 km^2^ and a population of 259,431 according to the 2006 census. It shares borders in the north with Benue State; in the south with Ovoko and Iheakpu-awka in Igbo-Eze South Local Government Area; in the east Amala and Obollo in Udenu Local Government Area; and with Kogi State in the West and partly in the north. In Igbo-Eze North Local Government Area, the people are predominantly traders, farmers and palm wine tappers. This region also boasts of many soothsayers, traditional medicine men and herbalists. The area is renowned for its palm wine production and African Traditional Religion (ATR). The overwhelming majority of the people live in rural settlements, where they mainly engage in subsistence agriculture and related activities.

### 2.2. Ethical Clearance

Ethical approval was procured from the State Ministry of Health, Enugu State (MH/MSD/REC18/007). Also, permission from the Health Department, Igbo-Eze North Local Government Council, was sought. Informed consent of the heads and medical personnel of health facilities and study participants of all ages were solicited to enable prompt recruitment.

### 2.3. Study Design

A cross-sectional community survey involving a multistage sampling procedure was used for this study. The first stage involved a purposive selection and stratification of the Local Government. A purposeful sampling method was adopted for the selection of the communities in the Local Government Area. The second stage involved the random selection of communities within different strata. At the community level, individuals were stratified according to age, sex and occupation. Selection of participants from the strata was based on random sampling using the lottery method. A rapid assessment method for onchocerciasis was carried out on the participants. The demographic information of the participants was captured using a questionnaire tool (see Supplementary Materials). The results from the laboratory and questionnaire investigation were used to estimate the prevalence and risks of onchocerciasis in the study area.

### 2.4. Sample Size Determination

The study population included residents of the study area who have lived in the Local Government Area for at least one year. A sample size of 384 individuals determined using the method devised by Sarmukaddam and Garad [5] from four (4) communities in the Local Government Area, namely, Aguibeje, Umuogboagu, Umuopu and Umuagama, was proposed, but 201 persons gave consent to participate in the study. The communities were selected following the Rapid Epidemiological Mapping of Onchocerciasis (REMO), which was devised by Ngoumou and Walsh [6] and states that communities selected should be located close to river banks.

### 2.5. Examination of Study Participants

**Figure 2:**
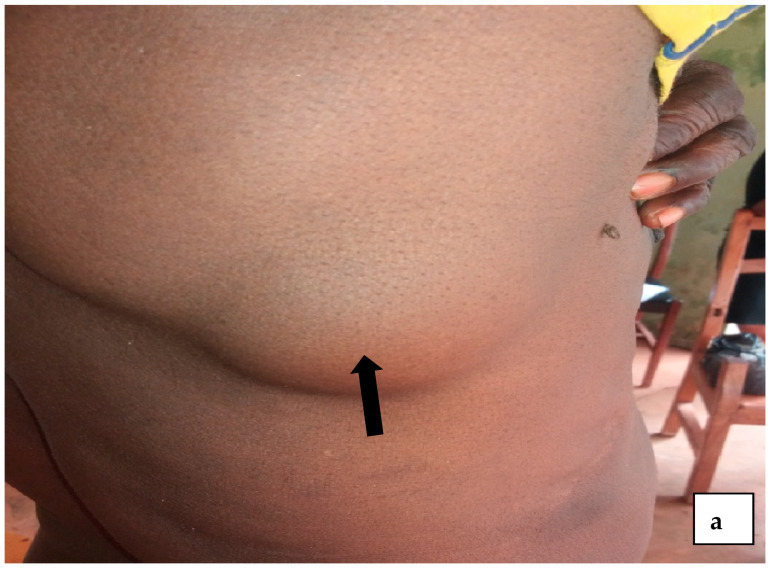

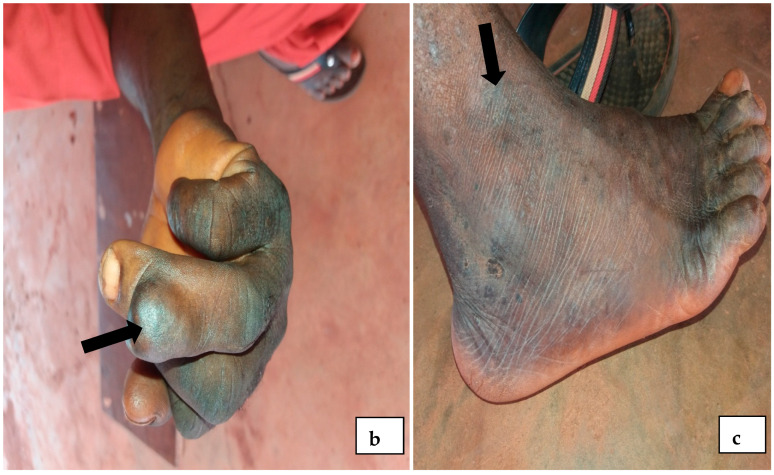
(**a**–**c**) A man from Aguibeje with both nodules and lizard skin.

**Figure 3:**
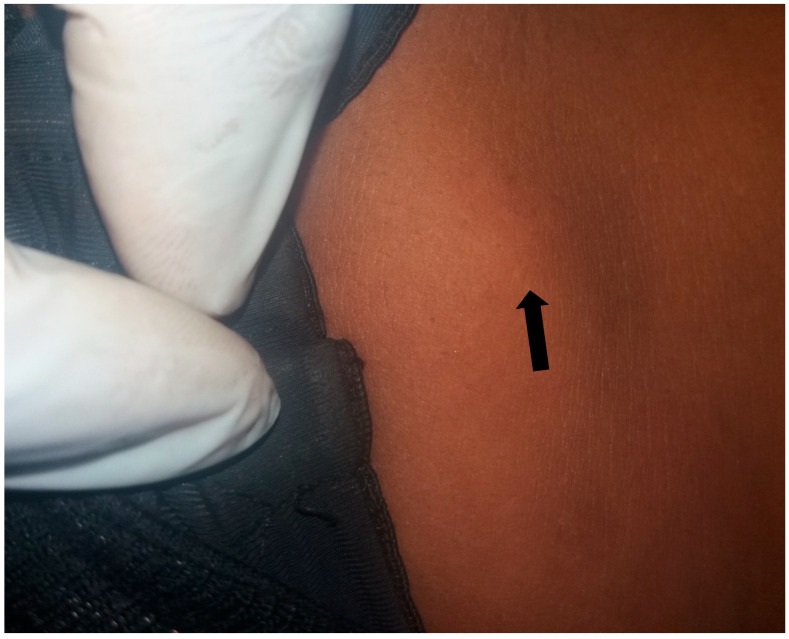
A woman from Umuogbuagu with a nodule.

**Figure 4:**
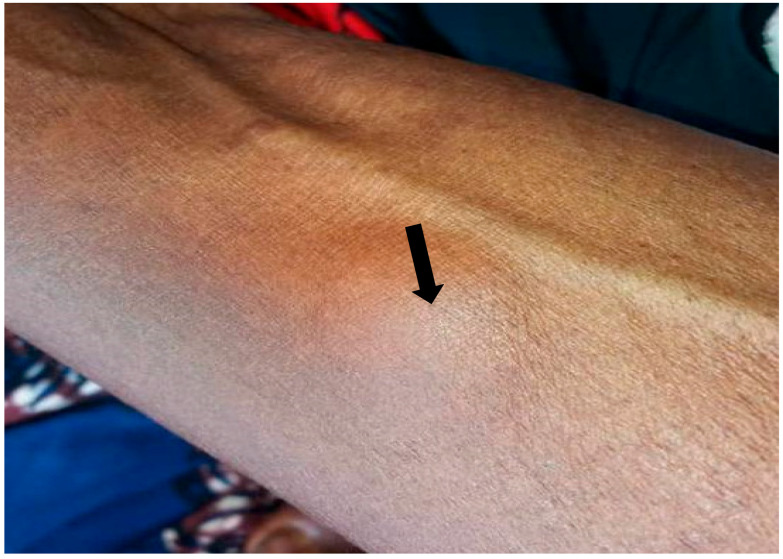
A woman from Umuopu with a nodule.

The participants were examined using a rapid assessment method (RAM) by trained medical personnel. A typical examination of each subject was carried out for obvious signs of onchocerciasis such as onchocercal depigmentation (leopard skin), lizard skin (Figure 2c), palpable subcutaneous nodules (Figure 2a,b, Figure 3 and Figure 4), hanging groins, pruritus as well as dermal fibrosis and atrophy [2]. Lizard skin, or lichenification, is characterized by thickened, dry and inelastic skin, often with a wrinkled appearance, while the leopard skin is characterized by patchy areas of depigmentation (loss of color), with small spots of normal skin remaining, mostly on the shins.

### 2.6. Administration of Questionnaire

A standardized, close-ended questionnaire was administered to each participant by an oral interview [7]. Demographic information, information on the domestic and peridomestic environment and information on personal activities outside of the peridomestic area that might be related to exposure to vector bites were included in the questionnaire. Parents and the guardians answered the questionnaire for their children or wards below 10 years of age.

### 2.7. Statistical Analysis

The data obtained from this study were entered into Microsoft Excel sheets, and all statistical analyses carried out using Statistical Product and Service Solutions (IBM SPSS, Chicago, IL, USA) software version 23.0. The prevalence of onchocerciasis among the study population was compared across the different study locations using Fisher’s exact test to determine differences in prevalences that were recorded by testing the association between categorical variables. Binary logistic regression was carried out to evaluate the risk factors associated with onchocerciasis among the residents of the study area. Differences in values were statistically significant at *p* < 0.05 (95% confidence interval).

## 3. Results

### 3.1. Prevalence of Onchocerciasis in Igbo-Eze North LGA

The overall prevalence of onchocerciasis in Igbo-Eze North Local Government Area is presented in Table 1. A total of 201 individuals were examined across the study area. From the result, it was observed that the overall prevalence of onchocerciasis was 3.5% (7/201). When onchocerciasis prevalences were compared for the communities, it was observed from the result that the differences were non-significant (*p* = 0.750).

The prevalence of onchocerciasis in Igbo-Eze North Local Government Area according to sex, age and occupation is presented (Table 2). From the result, sex-related onchocerciasis prevalences did not vary significantly (*p* = 0.699); however, it was more prevalent among the males (4.2%) than the females (3.1%). It was observed from the result that onchocerciasis prevalence varied non-significantly according to age (*p* = 0.493). Onchocerciasis was more prevalent among 30–39-year-old subjects (10.3%). In relation to occupation, the prevalence of onchocerciasis was significantly (*p* = 0.013) higher among farmers/fishermen, with a prevalence of 17.2% (5/29).

### 3.2. Risk Factors for Onchocerciasis in Igbo-Eze North LGA

The risk factors associated with onchocerciasis outcome in Igbo-Eze North LGA are presented in Table 3. The prediction from the result is that those that know the vector for onchocerciasis are more likely to have the disease than those without knowledge of the onchocerciasis vector (*p* = 0.034, OR = 29.000). Also, individuals that have houses close to the water body (*p* = 0.034, OR = 29.000) present a higher likelihood of *Onchocerca* infection and ultimately the disease. Other variables assessed did not add significantly (*p* > 0.05) to the prediction model.

## 4. Discussion

### 4.1. Prevalence of Onchocerciasis in Igbo-Eze North LGA

The overall prevalence of onchocerciasis was 3.5% (7/201) in the present study. The prevalence of onchocerciasis among the locations was comparable and did not show any significant (*p* > 0.05) differences, although Aguibege recorded the highest prevalence, while Umuogbuagu had the lowest. The present study had a much lower prevalence (3.5%) than an earlier study by Okoye et al. [3] in a neighboring community that showed a high onchocerciasis prevalence of 36.6%, with a prevalence of onchodermatitis of 46.2%. Another study conducted in Ebonyi State by Okonkwo et al. reported a higher prevalence of 33.3% [8]. The lower prevalence seen in this study may be due to public awareness campaigning and education on onchocerciasis in the area. Furthermore, the low prevalence could be attributed to the mass drug administration previously reported in the study area, although the re-assessment study of onchocerciasis after the 10-year mass mectizan chemotherapeutic intervention by Okoye et al. [3] revealed that despite the free distribution of the drug in the study area for a decade, onchocerciasis prevalence was still high.

Sex-related prevalence showed no significant (*p* > 0.05) differences; however, the present study implicated onchocerciasis infection to be higher among the males than females (Table 2). This corroborates earlier reports [9,10] that there exists no statistically significant difference between both sexes since both men and women engage equally in the activities that exposed them to the vectors and have the same living conditions. Also, exposure to various breeding sites of the vectors due to individuals’ poor environmental and unhygienic conditions might have accounted for the similar prevalence in both males and females. However, the majority of the respondents in our study were women, which may pose potential bias, although both males and females engage in socioeconomic activities (such as fetching water from the breeding site, farming, fishing and hunting), which could expose them alike to black fly bites. This agrees with earlier reports [3,11] and may be due to the relatively similar exposure of males and females. Our finding is also partly consistent with those of Njenga et al. [12] and Shiferaw et al. [13] that reported males to have a higher infection rate than females. They opined that males work longer in the farm and with a bare body in contrast to the females, thereby making them more prone to the bite of black flies. Also, results from studies by Basáñez and Boussinesq [14], Eyo et al. [15] and Ekpo et al. [4] reported that males had higher nodule prevalence than females. Altogether, we infer that differences in infection rate with regards to sex may be due to occupational exposure and the susceptibility of individuals as supported by previous authors [8].

In relation to age, participants aged 30–39 years had the highest onchocerciasis prevalence (*p* > 0.05) overall, followed by older subjects (≥50 years). This is similar to the findings of Oparaocha et al. [16], Usip et al. [9] and Okuliez et al. [17]. The high prevalence observed between 30 and 50 years might be due to the fact that older individuals have been exposed throughout their lives and that they are more exposed to the vectors because of their occupations, mostly as farmers in the fields, as opposed to the children who are attending school. Later in life, exposure continues during farming and other adult occupations.

Also, the farmers/fishermen had the highest onchocerciasis prevalence (*p* = 0.001). This corroborates the findings of Abdullahi et al. [18] and Okonkwo et al. [8] that field workers as well as people working close to the river are more predisposed to the bite of the infected black fly.

The small number of participants sampled per community is a limitation of our study given the fact that a smaller sample size predisposes estimates of infection prevalence by community to wide confidence intervals. Also, multivariable analysis was not conducted in this study as the analysis was limited to measuring bivariate associations and significance testing. However, this is a steppingstone to more advanced research using multivariable regression techniques.

### 4.2. Risk Factors for Onchocerciasis in Igbo-Eze North LGA

Our study implicated the proximity of the water body to houses in the risk for onchocerciasis in the study area. Knowledge of the onchocerciasis vector was high in the study area, which explains the low prevalence of onchocerciasis. In the control or elimination of a disease, the population involved must have prior knowledge of the disease for the control measure to be successfully implemented. Knowledge is essential for any eradication program to be achievable as poor knowledge leads to poor participation, leading to low coverage and persistence in transmission of the disease.

## 5. Conclusions

Onchocerciasis prevalence in Igbo-Eze North Local Government Area was low. In the study area prevalence differences were not dependent on sex or age but on occupation of the residents. Clinical manifestation of onchocerciasis showed debilitating potential among the infected individuals, with a great economic effect on their families and community at large. The findings from this study showed that there was some awareness regarding onchocerciasis among people in the study area. However, knowledge of the treatment was poor. It is possible that the prevalence and burden overtime of onchocerciasis will increase in the area. Urgent and targeted control measures especially among farmers should be undertaken for transmission blocking and elimination intervention. Awareness campaigning on the cause of onchocerciasis as well as mass drug administration should be intensified.

## Figures and Tables

**Table 1:** Overall prevalence of onchocerciasis in Igbo-Eze North LGA.

Community	Number Examined	Number Infected	Prevalence (%)
Aguibege	54	3	5.6
Umuopu	46	2	4.3
Umuogbuagu	52	1	1.9
Umuagama	49	1	2.0
**Overall**	**201**	**7**	**3.5**
F = 1.484, *df* = 3, *p* = 0.750			

Significant difference at *p* < 0.05; F, Fisher’s Exact test statistic; *df*, degree of freedom.

**Table 2:** Prevalence of onchocerciasis in Igbo-Eze North LGA according to sex, age and occupation.

Variable	Category	Number Examined	Number Infected	Prevalence (%)	*p*-Value
Sex	Male	71	3	4.2	0.699
	Female	130	4	3.1	
	**Total**	**201**	**7**	**3.5**	
Age (years)	0–9	9	0	0.0	0.493
	10–19	19	0	0.0	
	20–29	21	0	0.0	
	30–39	29	3	10.3	
	40–49	43	1	2.3	
	≥50	80	3	3.8	
	**Total**	**201**	**7**	**3.5**	
Occupation	None	8	0	0.0	0.013
	Schooling	25	0	0.0	
	Farmer/Fisherman	29	5	17.2	
	Civil Servant	17	0	0.0	
	Artisan	27	1	3.7	
	Trader	95	1	1.1	
	**Total**	**201**	**7**	**3.5**	

Significant difference at *p* < 0.05.

**Table 3:** Risk factors associated with onchocerciasis in Igbo-Eze North LGA.

Variable	Category	*df*	Odds Ratio (95% CI)	*p*-Value
Knowledge of onchocerciasis	Yes	1	0.744 (0.114–15.857)	0.814
	No		1	
Knowledge of possible causes of onchocerciasis	Charm	2	0.073 (0.674–277.123)	0.089
	Witchcraft		0.200 (0.240–104.147)	
	Genetic		1	
Seen onchocerciasis patient with signs/symptoms	Yes	1	0.517 (0.166–22.497)	0.599
	No		1	
Knowledge of onchocerciasis vector	Yes	1	29.000 (0.002–0.773)	0.034
	No		1	
Do you visit water bodies?	Yes	1	4.417 (0.018–2.884)	0.253
	No		1	
Proximity of water body to the house	Yes	1	29.000 (0.002–0.773)	0.034
	No		1	
Do you make use of mosquito nets?	Yes	1	0.452 (0.190–25.769)	0.526
	No		1	
What is your preferred drug of choice?	Hospital/Orthodox	1	1.105 (0.077–10.578)	0.936
	Traditional		1	

Significant odds ratio at *p* < 0.05.

## Data Availability

The original contributions presented in this study are included in the article/Supplementary Material. Further inquiries can be directed to the corresponding author.

## Supplementary Materials

The following supporting information can be downloaded at: https://www.mdpi.com/article/10.3390/tropicalmed10100285/s1, Questionnaire.
