# Supplementary material for: Epidemiology and Risks Survey of Onchocerca volvulus Infection in Igbo-Eze North Local Government Area, Enugu State, Nigeria
Source: Trop Med Infect Dis. 2025 Oct 6;10(10):285. doi: 10.3390/tropicalmed10100285 (PMC12568135; doi:10.3390/tropicalmed10100285)
Supplement: Supplementary file 1 [file tropicalmed-10-00285-s001.zip › tropicalmed-3733904-supplementary.pdf]

QUESTIONAIRE

PREVALENCE OF ONCHOCERCIASIS AMONG THE INDIVIDUALS IN THE SELECTED COMMUNITIES IN IGBO-EZE NORTH LGA, ENUGU STATE

Good day, please kindly respond to the questions honestly and to the best of your knowledge. This interview will take less than 30 minutes of your time. Do know that your answers will be treated with utmost confidentiality.

This study is aimed at investigating the prevalence of onchocerciasis among the individuals in the selected communities in Igbo-Eze North LGA, Enugu State.

BASIC INFORMATION

**Name of Community**	
**Respondents Identifier**	
**Date of Interview**	

SECTION A: SOCIO-DEMOGRAPHIC CHARACTERISTICS OF THE STUDY SUBJECTS

1. Age group: 0-9 [ ] 10-19 [ ] 20-29 [ ] 30-39 [ ] 40-49 [ ] ≥ 50 [ ]
2. Sex: Male [ ] Female [ ]
3. Marital status: Single [ ] Married [ ] Divorced [ ] Widow/widower [ ]
4. Occupation: Applicant [ ] Schooling [ ] civil servant [ ] Farmer/fisherman [ ] Artisan [ ] Trader [ ]
5. Educational status: Non-formal [ ] Primary [ ] Secondary [ ] Tertiary [ ]
6. Religion: Traditionalist [ ] Atheist [ ] Christianity [ ] Islam [ ]

SECTION B: CLINICAL CHARACTERISTICS FOR ONCHOCERCIASIS ASSESSMENT

7. Hardening of the skin? Yes [ ] No [ ]
8. Obstruction of vision? Yes [ ] No [ ]
9. Experience itching of skin? Yes [ ] No [ ]
10. How often do you itch? Rarely [ ] Sometimes [ ] Always [ ]
11. Deformation of skin? Yes [ ] No [ ]
12. Depigmentation of skin? Yes [ ] No [ ]
13. Presence of palpable nodules? Yes [ ] No [ ]

SECTION C: RISK FACTORS ASSOCIATED WITH ONCHOCERCIASIS

14. Knowledge of onchocerciasis? Yes [ ] No [ ]
15. Knowledge of possible causes of onchocerciasis? Charm [ ] Witchcraft [ ] Genetic [ ]
16. Seen onchocerciasis patient with signs/symptoms? Yes [ ] No [ ]
17. Knowledge of onchocerciasis vector? Yes [ ] No [ ]
18. Do you visit water bodies? Yes [ ] No [ ]
19. Proximity of water body to the house? Yes [ ] No [ ]
20. Do you make use of mosquito nets? Yes [ ] No [ ]
21. What is your preferred drug of choice? Hospital/Orthodox [ ] Traditional [ ]
